# Biological Effects of the Azaspiracid-Producing Dinoflagellate *Azadinium dexteroporum* in *Mytilus galloprovincialis* from the Mediterranean Sea

**DOI:** 10.3390/md17100595

**Published:** 2019-10-22

**Authors:** Maria Elisa Giuliani, Stefano Accoroni, Marica Mezzelani, Francesca Lugarini, Simone Bacchiocchi, Melania Siracusa, Tamara Tavoloni, Arianna Piersanti, Cecilia Totti, Francesco Regoli, Rachele Rossi, Adriana Zingone, Stefania Gorbi

**Affiliations:** 1 Dipartimento di Scienze della Vita e dell’Ambiente, Università Politecnica delle Marche, Via Brecce Bianche, 60131 Ancona, Italy; m.e.giuliani@staff.univpm.it (M.E.G.); s.accoroni@univpm.it (S.A.); m.mezzelani@pm.univpm.it (M.M.); francescalugarini91@gmail.com (F.L.); c.totti@univpm.it (C.T.); f.regoli@univpm.it (F.R.); 2 Istituto Zooprofilattico Sperimentale Umbria e Marche, Via Cupa di Posatora, 3, 60131 Ancona, Italy; s.bacchiocchi@izsum.it (S.B.); m.siracusa@izsum.it (M.S.); t.tavoloni@izsum.it (T.T.); a.piersanti@izsum.it (A.P.); 3 Istituto Zooprofilattico Sperimentale del Mezzogiorno, Via Salute 2, 80055 Portici (NA), Italy; rachele.rossi@izsmportici.it; 4 Dipartimento di Ecologia Marina Integrata, Stazione Zoologica Anton Dohrn, Villa Comunale, 80121 Napoli, Italy; zingone@szn.it

**Keywords:** azaspiracids, *Azadinium dexteroporum*, biotoxins, mussels, biomarkers, immune responses, storage lipids, genotoxicity

## Abstract

Azaspiracids (AZAs) are marine biotoxins including a variety of analogues. Recently, novel AZAs produced by the Mediterranean dinoflagellate *Azadinium dexteroporum* were discovered (AZA-54, AZA-55, 3-epi-AZA-7, AZA-56, AZA-57 and AZA-58) and their biological effects have not been investigated yet. This study aimed to identify the biological responses (biomarkers) induced in mussels *Mytilus galloprovincialis* after the bioaccumulation of AZAs from *A. dexteroporum*. Organisms were fed with *A. dexteroporum* for 21 days and subsequently subjected to a recovery period (normal diet) of 21 days. Exposed organisms accumulated AZA-54, 3-epi-AZA-7 and AZA-55, predominantly in the digestive gland. Mussels’ haemocytes showed inhibition of phagocytosis activity, modulation of the composition of haemocytic subpopulation and damage to lysosomal membranes; the digestive tissue displayed thinned tubule walls, consumption of storage lipids and accumulation of lipofuscin. Slight genotoxic damage was also observed. No clear occurrence of oxidative stress and alteration of nervous activity was detected in AZA-accumulating mussels. Most of the altered parameters returned to control levels after the recovery phase. The toxic effects detected in *M. galloprovincialis* demonstrate a clear biological impact of the AZAs produced by *A. dexteroporum*, and could be used as early indicators of contamination associated with the ingestion of seafood.

## 1. Introduction

Azaspiracids (AZAs) are lipophilic marine biotoxins causing azaspiracid poisoning (AZP) syndrome [1]. After the first case of intoxication (Killary Harbour, Ireland, 1995), AZAs were initially included among the diarrhetic shellfish poisoning (DSP) toxins, because they induced similar gastrointestinal symptoms [2]. Lately, the characterization of their chemical structure [3], along with the first evidence of a different mechanism of action [4], led to their classification as a stand-alone group [5,6]. 

To date, over 30 structural variants of AZAs have been isolated, either directly produced by phytoplankton species or biotransformed by accumulating shellfish [7,8,9,10]. Among them, the analogues AZA-1, AZA-2 and AZA-3 are the most relevant with regards to abundance and toxicity [11] and their total levels in the whole tissues of edible shellfish are regulated by the EU for human health protection [12], with a permitted level of 160 µg AZA equivalents kg^−1^ of mollusc flesh. The same value of AZAs is regulated at international level in the Standard for Live and Raw Bivalve Molluscs [13].

Since their discovery, AZAs have been detected in several marine mollusc and crustacean species from different coastal regions of Europe (i.e., Ireland, Norway, England, Spain, France, Denmark, Portugal and Sweden), Morocco and Canada, including mussels (*Mytilus galloprovincialis* and *Mytilus edulis*), oysters (*Crassostrea gigas* and *Ostrea edulis*), scallops (*Pecten maximus*), clams (*Tapes philippinarum*, *Ensis siliqua* and *Donas* spp.) and crabs (*Cancer pagurus*) [9 and references therein]. Recently, traces of AZAs were detected in *M. galloprovincialis* from the Italian coasts of the Mediterranean Sea [14]. 

Several dinoflagellates have been identified as primary AZA-producers: the first was *Azadinium spinosum*, followed by *Azadinium poporum*, *Amphidoma languida* and *Azadinium dexteroporum* [15,16,17]. The latter, collected in 2009 in the Gulf of Naples, is the most recently described species of the genus and one of the two *Azadinium* species recorded in the Mediterranean Sea, along with *A. poporum* [18]. Currently, *A. dexteroporum* represents the species producing the highest variety of AZAs, which does not include the typical AZA-1 and AZA-2, but AZA-35 plus six novel compounds: AZA-54, AZA-55, 3-epi-AZA-7, AZA-56, AZA-57 and AZA-58 [19]. 

**Figure 1:**
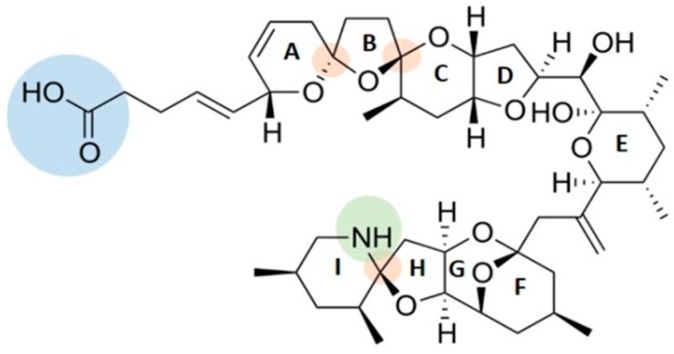
Azaspiracid (AZA)-1 structure with cyclic amine (aza group in green), a trispiro-group (orange) and a carboxylic acid group (blue).

AZAs structure is characterized by polyether rings with cyclic amine (aza group), a trispiro-group and a carboxylic acid group (Figure 1). 

At physiological pH (7.4), AZAs are present as zwitterions, neutral molecules with both positive and negative charges [1,3,11]. The properties of a both lipophilic and charged molecule ensure AZAs broad capabilities to cross cell membranes and interact with biological structures. Indeed, in vitro exposures of mammalian cell cultures to AZAs revealed a wide variety of effects, including F-actin decrease and cytoskeleton disorganization, increase of cytosolic calcium and cAMP, inhibition of neuronal bioelectric activity, activation of apoptotic pathways, hERG (human ether-a-go-go-related gene) potassium channel blockage, alteration in cell–cell adhesion, damages to mitochondria, generation of autophagosomes, ATP depletion, upregulation of proteins involved in energy metabolism and Golgi apparatus disruption [20,21,22,23,24,25,26,27,28,29]. In vivo toxicological studies in rodents revealed that oral administration of AZAs induce extensive damages to several organs (intestine, liver, spleen and thymus) and gastrointestinal symptoms (dilation and fluid accumulation in the small intestine, exfoliation of duodenal villi, infiltration of leukocytes), while intravenous or intraperitoneal injection caused neurotoxicity (sluggishness, respiratory difficulties, spasms, progressive paralysis), cardiotoxicity (arrhythmias, functional and structural heart damage) and cardiovascular problems (altered arterial blood pressure) [20,30,31,32,33]. Colman and colleagues demonstrated the teratogenic effect of AZA-1 in the embryos of the freshwater fish Japanese medaka *Oryzias latipes* [34], suggesting potential adverse outcome of AZAs in fish development, with consequent ecological impacts. Only few studies investigated the biological effects of AZAs in marine animal models in vivo, although they are the main targets and sources of AZA contamination [35,36]. To date, only *A. spinosum* and *A. poporum* were used in experimental conditions, revealing a negative effect on feeding behaviour of mussels [35] and no effects on antioxidant enzyme activities in mussel and scallop tissues [36].

Several studies have underlined the complexity of AZAs effects, since they affect multiple cellular targets and induce different responses depending on the experimental models [20,25,37]. Such complexity has hampered an unambiguous identification of the primary biological target of AZAs, and a clear elucidation of the mechanisms of AZA toxicity. In addition, various AZA analogues seem to have a different toxicity, probably due to their specific molecular structures [19,38]. In such context, the “exceptional diversity” [19] of AZAs produced by the Mediterranean *A. dexteroporum* strain is of particular interest, considering also that none of the AZAs it produces are currently regulated in the EU. In particular, toxicological effects of AZAs produced by *A. dexteroporum* are relevant for endemic Mediterranean bivalves, such the mussel *M. galloprovincialis*. 

The present study represents the first experimental approach with *A. dexteroporum* as source of AZAs for an in vivo laboratory exposure. Mussels *M. galloprovincialis* were fed with the AZA-producing dinoflagellate for 21 days, followed by a recovery period. The experiment was planned to observe early effects occurring at low AZAs concentrations, and a wide variety of cellular and biochemical responses (biomarkers) were investigated, including immunological and antioxidant functions, histological changes, alterations of lipid metabolism, alterations of acetylcholinesterase activities and genotoxicity. Bioaccumulation of AZAs was measured in exposed mussel, and the toxin profile of the strain used was compared with that of mussel tissues and biodeposits (mussel faeces and pseudofaeces) analysed throughout the exposure, using liquid chromatography coupled with tandem mass spectrometry (LC-MS/MS). The general aim of the study was to define potential biomarkers for an early detection of AZAs exposure in mussels and provide new insights on potential mechanisms of AZAs toxicity. 

## 2. Results

### 2.1. AZAs Profile in A. dexteroporum, Mussel Tissues and Biodeposits

**Figure 2:**
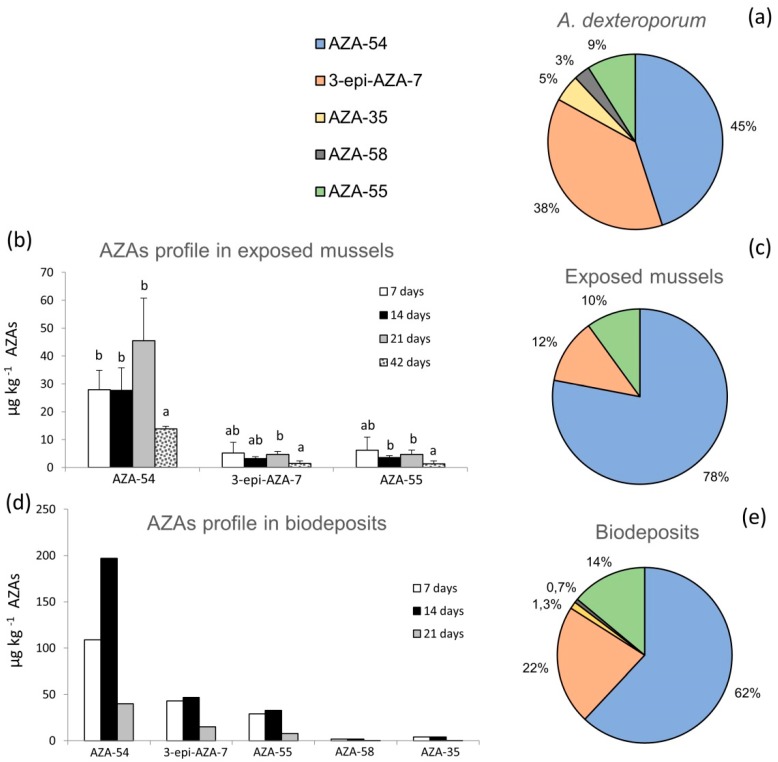
AZAs toxin profile: (**a**) relative abundance of AZAs in *Azadinium dexteroporum* Mediterranean strain; (**b**) AZAs bioaccumulation in whole tissue of mussels fed on *A. dexteroporum* after 7, 14, 21 days of exposure and after 21 days of recovery (42 days) (mean ± standard deviation of three experimental replicates); (**c**) AZAs toxin profile in mussel whole tissue during the 21 days of exposure (mean of the whole period); (**d**) AZAs toxin profile in biodeposits produced by mussels fed on *A. dexteroporum* during the exposure (7, 14, 21 days); (**e**) AZAs toxin profile in biodeposits during the 21 days of exposure (mean of the whole period). Different letters on top of the histogram bars indicate significant differences between means, within the same AZA analogue.

The LC-MS/MS analysis of the *A. dexteroporum* strain used in laboratory experiments highlighted five AZA analogues in the toxic profile, with a total AZAs cell quota of 19 fg cell^−1^ (as sum of all analogues’ concentrations). The AZA-54 and 3-epi-AZA-7 analogues were the most abundant (45% and 38% of total AZAs concentration, respectively), followed by AZA-55 (9%), AZA-35 (5%) and AZA-58 (3%) (Figure 2a). No AZAs were detected in the algal culture medium.

Exposed mussels showed the bioaccumulation of three azaspiracids, AZA-54, 3-epi-AZA-7 and AZA-55, reaching a total AZAs concentration of 56 µg kg^−1^ at the end of the exposure experiment (i.e., 21 days). Bioaccumulation of AZA-54 increased to a maximum value of 46 ± 15 µg kg^−1^ after 21 exposure days and decreased down to 14 ± 1 µg kg^−1^, after 21 days of recovery (Figure 2b). Lower concentrations were measured for 3-epi-AZA-7 and AZA-55, with limited variations during the exposure phases (Figure 2b). AZA-35 and AZA-58 were always below the limit of detection (LOD), in all analysed mussels samples. AZAs concentrations, averaged over the 21 days of exposure, revealed that AZA-54 was the predominant analogue (78%), followed by 3-epi-AZA-7 (12%) and AZA-55 (10%) (Figure 2c). Of the total, 75% of AZAs accumulated in the digestive gland of the exposed mussels, while the remaining 25% was distributed in the rest of tissues (data not shown).

The toxic profile of the biodeposits (faeces and pseudofaeces) produced by exposed mussels included five AZA analogues, which increased during 14 days of exposure and then decreased at 21 days (Figure 2d). Considering the mean within the 21 days of exposure, AZA-54 was the most abundant analogue in biodeposits (62%), followed by 3-epi-AZA-7 (22%), AZA-55 (14%), AZA-35 (1.3%) and AZA-58 (0.7%) (Figure 2e).

### 2.2. Biomarkers in Mussel Tissues

Biomarkers were evaluated in mussels exposed to the dinoflagellate *A. dexteroporum* and compared to mussels fed on a control diet. 

**Figure 3:**
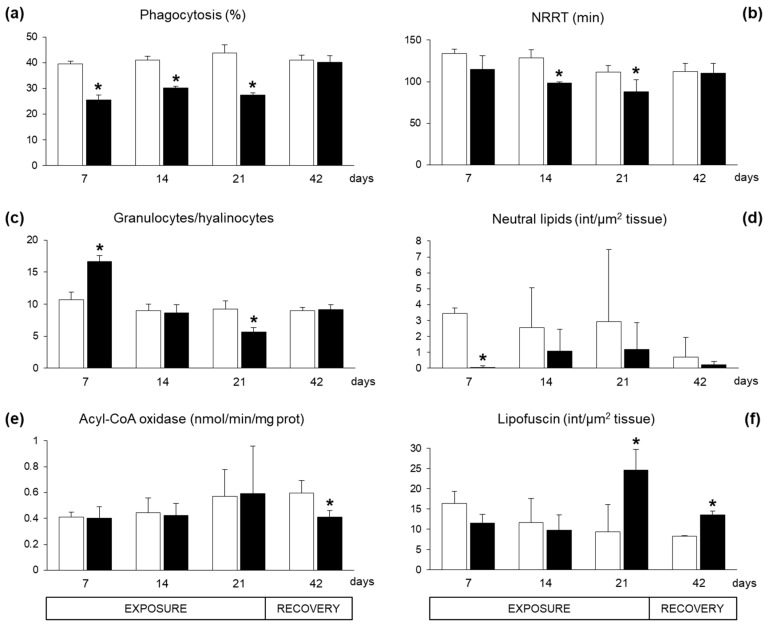
Immunological and lipid metabolism-related parameters measured in mussels *Mytilus galloprovincialis* exposed to *Azadinium dexteroporum* (black bars) and control mussels (white bars). (**a**) Phagocytosis activity; (**b**) lysosomal membrane stability, measured as neutral red retention time (NRRT); (**c**) granulocyte/hyalinocyte ratio; (**d**) neutral lipid content; (**e**) activity of acyl-CoA oxidase; (**f**) lipofuscin content. Values are expressed as mean ± st. dev. (n = 5). Asterisks represent statistically significant differences between means, in exposed and control groups (*p* < 0.05).

Immunological responses in haemocytes of exposed mussels revealed a significant inhibition of phagocytosis capacity (Figure 3a). The lysosome membrane stability, as neutral red retention time (NRRT), showed a decreasing trend with statistically significant differences after 14 and 21 exposure days (Figure 3b). The proportion of haemocytic subpopulation, granulocyte/hyalinocyte ratio, displayed a biphasic time-dependent modulation in exposed mussels, with a statistically significant increase after seven days followed by a strong decrease after 21 days (Figure 3c). After 21 days of recovery, exposed and control organisms showed comparable levels of immunological parameters (Figure 3a–c).

The content of neutral lipids in the digestive gland cells showed a general reduction in exposed mussels, with a statistically significant difference only after seven exposure days (Figure 3d). The enzymatic activity of the acyl-CoA oxidase (ACOX), involved in peroxisomal β-oxidation of fatty acids, was not altered in the digestive tissues during the exposure period, but showed a statistically significant inhibition after 21 days of recovery (Figure 3e). The accumulation of lipofuscin, a non-degradable polymer derived from incomplete oxidation of lipids and proteins in the lysosomes, significantly increased in digestive gland cells of organisms exposed to *A. dexteroporum* for 21 days. This parameter remained significantly higher than in controls also after the recovery phase (Figure 3f).

**Figure 4:**
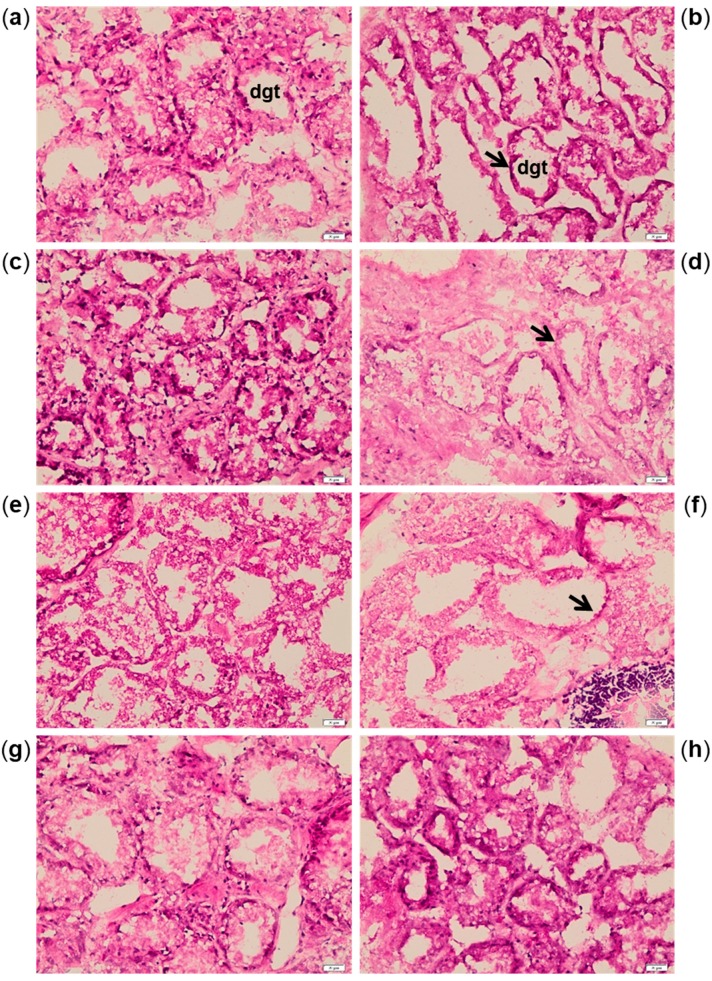
Haematoxylin–eosin staining on digestive gland tubules (dgt) of control mussels (**a**,**c**,**e**,**g**) and mussels exposed to *Azadinium dexteroporum* (**b**,**d**,**f**,**h**). The pictures are representative of samples from 7 (**a**,**b**), 14 (**c**,**d**) and 21 (**e**,**f**) days of exposure and 21 days of recovery (**g**,**h**). The black arrows indicate the thinned digestive tubules in exposed mussels. Magnification: 200×.

Histological alterations were highlighted in digestive gland sections after 7, 14 and 21 days of exposure to *A. dexteroporum*, with reduction of digestive tubule thickness and consequent increase of the lumen surface (Figure 4a–f). The digestive tissue of exposed mussels returned to a normal morphological structure after 21 days of recovery (Figure 4g–h).

**Figure 5:**
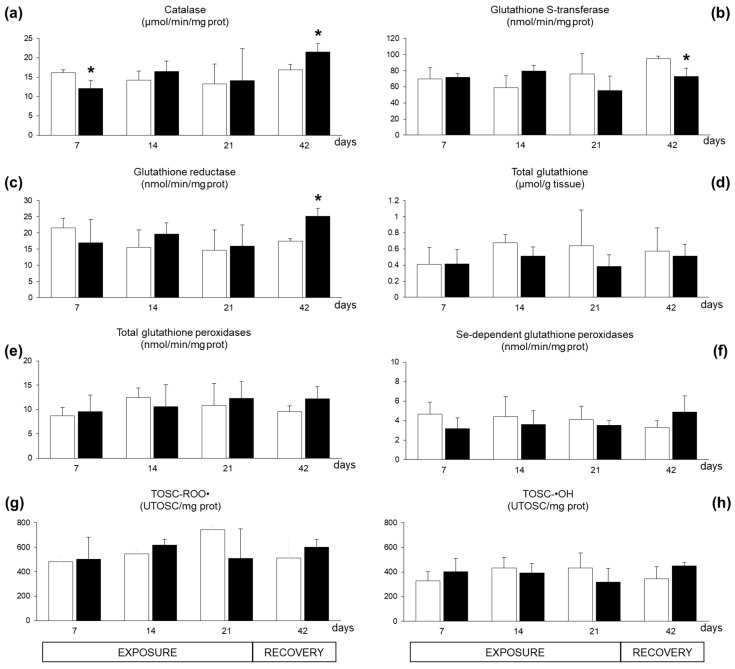
Antioxidant parameters analysed in *Mytilus galloprovincialis* exposed to *Azadinium dexteroporum* (black bars) and control mussels (white bars). (**a**) Catalase activity; (**b**) glutathione S-transferases activity; (**c**) glutathione reductase activity; (**d**) total glutathione level; (**e**) activity of total glutathione peroxidases; (**f**) activity of Se-dependent glutathione peroxidases; (**g**) total oxyradical scavenging capacity against peroxyl radicals (TOSC-ROO^●^); (**h**) total oxyradical scavenging capacity against hydroxyl radicals (TOSC-^●^OH). Values are expressed as mean ± st. dev. (n = 5). Asterisks represent statistically significant differences between exposed and control groups (*p* < 0.05).

Limited variations were observed for antioxidant responses in digestive glands. A seven-day exposure to *A. dexteroporum* significantly inhibited the enzymatic activity of catalase, which was later re-established and even increased after the recovery period (Figure 5a); at this time, glutathione S-transferase activity showed a significant inhibition while glutathione reductase activity increased (Figure 5b–c). No significant variations were observed for levels of total glutathione, glutathione peroxidase activities and total antioxidant scavenging capacity toward peroxyl and hydroxyl radicals (Figure 5d–h).

**Figure 6:**
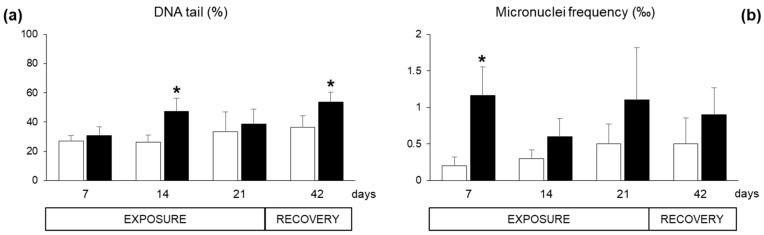
Genotoxic parameters measured in the haemocytes of *Mytilus galloprovincialis* exposed to *Azadinium dexteroporum* (black bars) and control mussels (white bars). (**a**) DNA fragmentation, assessed through the Comet assay and expressed as DNA percentage in the tail; (**b**) micronuclei frequency. Values are expressed as mean ± st. dev. (n = 5). Asterisks represent statistically significant differences within exposed and control groups (*p* < 0.05).

Specific responses of neurotoxicity, measured as acetylcholinesterase (AChE) activity in the haemocytes, were not altered during the exposure period (data not shown). Concerning genotoxic damage, the Comet assay revealed DNA fragmentation in haemocytes of exposed organisms after 14 days and at the end of the recovery, while the results were not statistically different at day 7 and 21 (Figure 6a). The micronuclei frequency was enhanced both during the exposure period and after the recovery, with statistically significant difference only at day seven (Figure 6b).

## 3. Discussion

The known complexity of AZA effects, which depends on different experimental models and is related to specific analogue structures, is growing along with the detection of novel AZA variants [7,19]. This study has investigated the biological effects induced in mussels after bioaccumulation of novel AZAs produced by a Mediterranean strain of the dinoflagellate: *A. dexteroporum*. We demonstrated that mussels fed on *A. dexteroporum* accumulate different AZA analogues, namely AZA-54, 3-epi-AZA-7 and AZA-55, in different percentages, with a maximum total AZAs concentrations of 56 µg kg^−1^ whole tissues, which is well below the maximum permitted level in the European regulation of 160 µg kg^−1^ [10].

The predominance of AZA-54 in mussel tissues reflects its relative abundance in the algal toxin profile, while its increasing bioaccumulation during the exposure time matched the lower percentage of this analogue in biodeposits (62%) compared to tissues (78%) indicating a high retention rate of this molecule. The percentage of 3-epi-AZA-7 in biodeposits (22%), higher than in tissues (12%), was compatible with its constant level in mussels during the exposure time and indicated a low accumulation rate of this compound despite its high level in algal cells (38%). AZA-55 showed a similar percentage in the algal cells, mussel tissues and biodeposits, suggesting compensation between accumulation and excretion/metabolization processes. Although with low percentages, AZA-35 and AZA-58 were present in algal cells and biodeposits but not in exposed mussels. A differential accumulation of AZA analogues (AZA-1, AZA-2) in exposed mussels was already reported by Jauffrais and colleagues, due to a different affinity for mussel tissues, most likely related to the molecular properties of these compounds [39]. 

One of the earliest alterations detected in mussels treated with *A. dexteroporum* was the inhibition of phagocytosis capacity of haemocytes already after seven exposure days. Such a result confirms previous studies on cultured cells that demonstrated that AZA-1 inhibits endocytosis [25,40] by causing the accumulation of the lysosomal protein cathepsin D [41], observed also in the digestive glands of AZA-contaminated mussels [42]. The loss of lysosomal membrane integrity in haemocytes further suggests that these organelles are an early cellular target of AZAs. A key role of lysosomes in AZA-induced toxicity was proposed also by Ferron and colleagues in cultured cells exposed to AZA-1 [43], similarly to other purified biotoxins like yessotoxins [44], or toxic microalgae like *Ostreopsis* cf. *ovata* [45] and *Alexandrium minutum* [46]. 

Since the haemocytes have a central role in mussel immune system, their impairment has direct implications on the health conditions of the organisms. Haemocyte populations include granulocytes (cells with phagocytic activity involved in the response to infections) and hyalinocytes (with supposed role in coagulation and encapsulation processes) [45,47] and their proportion (G/H ratio) can vary upon exposure to environmental stress conditions [45,48,49]. G/H ratio increased early in haemocytes of mussels fed on *A. dexteroporum*, suggesting an activation of the innate immunity as the first line of defence, which, however, did not appear to be functional, given the simultaneous inhibition of phagocytosis. The later reduction of G/H ratio (21 days) highlighted a biphasic response of this biomarker, possibly reflecting an impairment of immune functions in the presence of *A. dexteroporum*. Adverse effects of AZAs were documented also in mice immune system, where necrosis and reduced numbers of non-granulocytes and lymphocytes were observed in lymphoid organs after 4–24 h oral administration of high AZA doses (500–900 µg/kg) [30,50]. Based on previous studies [25,30,40,41,50], AZAs could induce an overall immunosuppressive effect, initially as a direct mechanism through inhibition of phagocytosis, and later (or at high doses) as an alteration of immune cells and organs. Similar effects would be caused by a primary inhibition of endocytosis with consequent alterations to lysosomes that would contribute to the impairment of immune and gastrointestinal tissues [41]. The immunomodulatory action of several harmful microalgae or biotoxins was previously highlighted in bivalves, either as immunostimulation (e.g., immune cell recruitment, activation of innate immunity, stimulation of phagocytosis) or as immunosuppression (damage to immune organs, apoptosis after toxin uptake, reduced haemocyte viability, inhibition of phagocytosis), depending on the algal species [45,46,51,52,53,54,55,56,57,58]. 

AZAs detected in this study were predominantly distributed in the digestive gland (75%), confirming this tissue as one of the main targets of lipophilic biotoxins including AZAs [59,60]. The accumulation of AZAs also caused a thinning of the digestive tubules wall and dilation of the lumen. Histopathological changes have been already described in bivalves exposed to *A. spinosum* [39] or other toxic microalgae (e.g., *Prorocentrum rhathymum*, *O.* cf. *ovata*, *Gymnodinium catenatum* and *Prorocentrum lima*) [45,58,61,62,63], and in mice, where AZAs caused erosion and shortening of intestinal villi [30,50]. However, a direct role of AZAs on digestive tissue morphology is still controversial, since no structural damage to intestinal cells was demonstrated after in vitro treatment with these biotoxins [29]. Since the atrophy of digestive tubules is a typical symptom of reduced feeding or starvation in bivalves [61], a similar mechanism may be hypothesised in the mussels exposed to *A. dexteroporum*, as often observed in bivalves exposed to toxic microalgae [35,64,65]. The above-mentioned effects of AZAs on endocytosis/phagocytosis could be responsible for an altered cellular assimilation of nutrients, while a disturbance of feeding activity seemed unlikely, since *A. dexteroporum* cells were quickly filtered by mussels and then cleared from water in 2 h after the inoculation (data not shown). A different behaviour was instead demonstrated for *A. spinosum*, which had a negative effect on feeding activity of *M. edulis* [35]. 

The consumption of neutral lipids in the digestive cells of mussels fed on *A. dexteroporum* corroborates an impairment in nutrient assimilation, since these reserves can be recycled for energy production [66], as also observed in bivalves exposed to the toxic *Alexandrium minutum* [67] or *O.* cf. *ovata* [41]. On the contrary, AZAs caused accumulation of fat droplets in mice liver [30]. An involvement of autophagy can be hypothesized in digestive glands accumulating *A. dexteroporum*-AZAs, consistently with the regulation of neutral lipid metabolism upon food restriction [66] and the observation of autophagosomes in the cytoplasm of AZA-1 treated intestinal cells [29]. Autophagy has been also associated with destabilization of lysosomal membranes and accumulation of lipofuscin in the lysosomes [68,69], which were both observed in the digestive gland of *A. dexteroporum*-exposed mussels. Lipofuscin is a non-degradable polymer resulting from incomplete autophagic degradation of lipids and proteins: it accumulates during intense lysosomal activity, resulting from enhanced lipid peroxidation processes [68,69] and disturbance of lipid metabolism caused by nutritional deficiency [53]. We can thus hypothesize that a nutritional deprivation after exposure to *A. dexteroporum* may have activated autophagic mechanisms with consequent consumption of neutral lipids, observed already after seven exposure days; the increased lysosomal activity would have damaged lysosomal membranes (14 days) and induced the accumulation of lipofuscin (21 days). Nevertheless, given the lack of statistical difference observed on neutral lipids’ results at day 14 and day 21, further analyses are needed to validate this hypothesis. 

Our results on the peroxisomal activity of ACOX exclude the possibility that AZAs act as peroxisomal proliferators, as other algal metabolites do [70,71,72], since no statistical differences were reported for this biomarker, except for the recovery phase. 

Slight changes of antioxidants and total antioxidant capacity revealed a limited role of AZAs in the activation of pro-oxidant mechanisms in digestive glands of mussels exposed to *A. dexteroporum*. This is in accordance with a previous study, showing limited variations of antioxidants in *M. galloprovincialis* exposed to *A. poporum* [36]. 

The possibility that AZAs act on mussel nervous activity was evaluated in this study through the activity of AChE, involved in nerve impulse transmission. While in vivo and in vitro studies in mammalian models (mice and human neuronal cultures, respectively) associated AZA-1 and AZA-2 exposure to neurotoxicity [20,24,26], no evidence of a similar activity were obtained in mussels, which showed no variations of AChE during the exposure to *A. dexteroporum*-AZAs.

Although the genotoxic effects of AZAs are poorly investigated, AZA-1, AZA-2 and AZA-3 did not induce DNA double-strand breaks in human cell lines [43]. Conversely, an increase of DNA fragmentation was observed in the haemocytes of mussels exposed to *A. dexteroporum* after 14 exposure days, even though such an increase was not significant at day 7 and 21. The genotoxic damage to the haemocytes may represent a further impairment of the immune system. The genotoxicity was evidenced even at a chromosomal level already after seven days, with increased frequency of micronuclei, small DNA portions that separate from the nucleus at the end of the cellular division. The velocity and the intensity of this response, usually slower than the DNA fragmentation processes, together with the variations of haemocyte subpopulations (G/H ratio) observed at seven days, may indicate that the enhanced frequency of micronuclei is more probably related to the increase of cell turnover rate rather than to a direct genotoxic effect induced by AZAs. In fact, no differences were observed for longer periods (14 and 21 days).

Upon return to a normal feeding for a three-week period, most of the biological parameters altered during *A. dexteroporum* exposure (i.e., immune responses, digestive gland histology, neutral lipid content, micronuclei frequency) returned comparable to those of control organisms, demonstrating that the organisms were able to recover from the major alterations. However, a few biological effects were still observable after the depuration phase. Among these, the modulation of some antioxidant enzymes (catalase (CAT), glutathione S-transferases (GST) and glutathione reductase (GR)), the persistence of lipofuscin granules in the digestive glands and a certain fragmentation of DNA in the haemocytes, overall suggest a potential activation of oxidative metabolism during the excretion phase rather than during the exposure period. Such late effects may be also caused by the low but measurable amount of AZAs still present after the depuration in the tissues of mussels previously fed on *A. dexteroporum*. A similar persistence of AZAs after a recovery period was described in *M. edulis* fed on *A. spinosum*, and a biphasic detoxification kinetic, characterized by a rapid initial phase followed by a slower one, was proposed for AZAs, as already described for other toxins [39]. Such a slow detoxification process enabled the observation of effects at a very low level of AZAs bioaccumulation. 

## 4. Materials and Methods 

### 4.1. Algal Cultures

The strain SZN-B848 of *A. dexteroporum* was isolated from a water sample collected on May 11, 2010 at the LTER-MC (Long-Term Ecological Research Station MareChiara) in the Gulf of Naples [17]. Cells were cultured at 21 ± 0.1 °C under a 12:12 h L:D (light:dark) photoperiod and an irradiance of 90–100 µmol m^−2^ s^−1^, in modified f/4 medium Si-free, prepared by adding macronutrients at a Si-free f/4 medium [73] and selenium to filtered and autoclaved natural seawater (salinity 35). Trace metals, iron, vitamins (H, B1 and B12) and HEPES (4-(2-hydroxyethyl)-1-piperazineethanesulfonic acid, Sigma-Aldrich Co., St. Louis, MO, USA) pH 7.1 were added at levels corresponding to f/2 medium. 

The diatom *Skeletonema marinoi* was cultured at the same physical conditions in f/2 medium and used in the controls, as it is a common species occurring in the phytoplankton communities of northern Adriatic Sea [74] and already used in aquaculture applications [45]. 

The abundance of both microalgae was estimated using an inverted microscope (Zeiss Axiovert 135) equipped with phase contrast, at 400× magnification. Subsamples (1 mL) were settled in counting chambers after homogenization, according to the Utermöhl sedimentation method [75]. Counting was performed on 1–2 transects, found to give a reliable and repeatable cell number which was expressed as number of cells L^−1^. Microalgae were cultured to the stationary phase (2.6 × 10^5^ cells mL^−1^) before inoculation in the following experiments.

### 4.2. Mussel Exposure 

*Mytilus galloprovincialis* specimens (5 ± 1 cm length) were obtained from classified shellfish production sites [76] located along the Marche Coast (NW Adriatic Sea) in March 2016. After cleaning the shell to remove epibionts, 360 organisms were distributed in 20 L tanks (60 organisms per tank), acclimatized for 10 days to laboratory conditions with synthetic water (Instant Ocean sea salt) at 28–30 g L^−1^ salinity and 18 ± 1 °C and daily fed with *Skeletonema marinoi*. 

The AZA content in mussels before the experiment (time 0) was below the limit of quantification (LOQ). During the exposure period (21 days), control mussels (three tank replicates) were fed with *S. marinoi* (1 × 10^6^ cells L^−1^), while exposed mussels (three tank replicates) were fed with *A. dexteroporum* (1 × 10^4^ cells mL^−1^) and *S. marinoi* (1 × 10^6^ cell L^−1^). After the exposure period, mussels were maintained at the same conditions, without addition of *A. dexteroporum,* for 21 days more (recovery period). Water was renewed and microalgae added daily. Control and exposed organisms were sampled after 7, 14, 21 and 42 days from the beginning of the experiment; after each sample collection, water volume was restored, maintaining a mussel density of three organisms L^−1^. 

The concentration of *A. dexteroporum* in the experiment tanks was based on previous laboratory experiments with another *Azadinium* species [34] and can be considered environmentally realistic, since it reflects the highest abundances ever observed for this species of this genus, which attained 9 × 10^6^ cells L^−1^ (Argentine Sea [77]). Nevertheless, in the Adriatic Sea, a bloom event (10^6^ cells L^−1^) of cf. *Azadinium* was reported only in 2016 [78].

For chemical analysis of AZA compounds, whole tissues of five exposed mussels were sampled from each of the three tanks and pooled (n = 3) to obtain a proper amount of material for chemical analysis. In addition, five digestive glands (DGs) of exposed mussels were dissected and pooled from each of the three tanks (n = 3) for AZAs tissue distribution analysis. DG accounted for 16% of the total flesh mussel weight.

Biodeposits (faeces and pseudofaeces) were sampled from each tank at each exposure time (7, 14 and 21 days) before water renewal, using a Pasteur pipette [79], and pooled in order to obtain enough material for chemical analysis (nearly 2 g). The samples were centrifuged for 30 min at 9500 × *g*; the pellet was washed and resuspended twice with synthetic seawater (Instant Ocean sea salt) at 28–30 g L^−1^ salinity, and then frozen at −20 °C for subsequent chemical analyses. 

For biological analyses, five pools of DGs and haemolymph were obtained from 15 organisms (five organisms from each of the three tank replicates), for both the control and exposed group. DGs were dissected, frozen in liquid nitrogen and stored at −80 °C for biochemical and histological analyses (n = 5). The haemolymph, collected from the adductor muscle, was in part frozen in liquid nitrogen, stored at −80 °C for the analysis of acetylcholinesterase activity, and in part immediately used for the analysis of immunological parameters and of genotoxic damage (n = 5). 

### 4.3. Chemical Analyses 

#### 4.3.1. Chemicals and Standards

All chemicals were of analytical reagent grade: acetonitrile and 25% ammonia (LC-MS grade), methanol (HPLC grade), sodium hydroxide, 37% hydrochloric acid, acetone and dichloromethane (analysis grade) (Sigma-Aldrich Co., St. Louis, MO, USA). Water was MilliQ grade (18.2 MΩ cm) (Millipore Ltd., Bedford, MA, USA). Certified reference material for azaspiracid 1 (AZA-1) was purchased from the Institute of Biotoxin Metrology at the National Research Council of Canada (NRCC, Halifax, Nova Scotia, Canada). The primary standard was diluted to prepare concentrated stock standard solutions prior to further dilution in solvent to obtain the calibrants. 

#### 4.3.2. AZAs Extraction from *A. dexteroporum*

AZAs were extracted from *A. dexteroporum* using the modified procedure described by Jauffrais et al. [80]. An aliquot of 20 mL (5.3 × 10^6^ cells) of *A. dexteroporum* cultures was centrifuged for 20 min at 2500 × *g* (4 °C). The culture supernatant was collected for liquid–liquid extraction as described below. The pellet was re-suspended in 1 mL of acetone, vortex-mixed for 1 min and bath-sonicated for 10 min. After sonication, the sample was centrifuged for 10 min at 2500 × *g* (4 °C) and the supernatant transferred to a 5 mL glass tube. Pellet extraction was repeated three times, the supernatants combined and evaporated under nitrogen stream at 35 °C. The residue was dissolved in 1 mL of methanol and filtered through a 0.2-µm syringe filter (Minisart, Sartorius, Germany) before LC-MS/MS analysis. The supernatant obtained from centrifugation of *A. dexteroporum* culture was added with 2 mL of dichloromethane, vortex-mixed for 1 min and the organic phase transferred to a 15-mL glass tube. Supernatant extraction was repeated three times, the dichloromethane extracts combined and evaporated under nitrogen stream at 35 °C. The residue was dissolved in 1 mL of methanol and filtered before LC-MS/MS analysis.

#### 4.3.3. AZAs Extraction from Mussels (Whole Flesh, Digestive Gland) and Biodeposits

The official EU-RL (European Union Reference Laboratory) LC-MS/MS method [81,82] was adopted. Shellfish homogenate (2.00 ± 0.05 g) was extracted twice with 9 mL of methanol. After the first methanol addition, the sample was vortex-mixed for 3 min at 2000 rpm with a Multi Reax (Heidolph, Germany), the extract centrifuged for 10 min at 2000 × *g* and the supernatant transferred to a 20 mL volumetric flask. During the second methanol addition, the mixture was homogenized with an Ultra Turrax T25 mixer (IKA Works, Wilmington, NC, USA) for 1 min at 10,000 rpm, the extract centrifuged (10 min at 2000 × *g*), the supernatant was combined with the first extract and the whole extraction volume brought to final volume of 20 mL with methanol. An aliquot of the methanolic extract was filtered through a 0.2-µm syringe filter (Minisart, Sartorius, Germany) and submitted to AZAs determination. 

#### 4.3.4. LC-MS/MS Analyses 

LC-MS/MS analyses were performed using a hybrid triple–quadrupole/linear ion trap 3200 QTRAP mass spectrometer (AB Sciex, Darmstadt, Germany) equipped with a Turbo V source and an electrospray ionization (ESI) probe. The mass spectrometer was coupled to an Agilent 1200 HPLC (Palo Alto, CA, USA), equipped with solvent reservoir, in-line degasser, quaternary pump, refrigerated autosampler and column oven. 

AZA analyses were carried out using an X-BridgeTM C18 column (3 mm × 150 mm, 5 µm particle size; Waters, Milford, MA, USA) thermostated at 40 °C. Mobile phase A was H_2_O and B acetonitrile/H_2_O (90:10, v/v), both containing ammonium hydroxide (0.05% v/v) (pH 11). Chromatographic conditions adopted are described in Table S1. 

Infusion experiments were performed using certified reference material AZA-1 to set turbo IonSpray source parameters (Table S2). AZAs were monitored in the samples by precursor ion scanning (PIS) experiments carried out in positive ion mode, scanning m/z 672, m/z 670, m/z 658 fragments precursors in the mass range m/z 200–1000, using collision energy = 55 eV. To confirm and quantify AZAs content, multiple reaction monitoring (MRM) experiments were performed in positive ion mode by selecting two product ions for each toxin. Mass transitions and collision energy used are shown in Table S3. Quantitation was performed by external calibration, assuming an equi-molar response. AZA-1 was used for indirect quantification of all AZAs. Method performances were evaluated in terms of linearity of the response (R^2^ = correlation coefficient, RF = response factor), sensitivity (limit of detection, LOD and limit of quantification, LOQ), accuracy (R% = recovery) and precision as intra-day repeatability (intra-day relative standard deviation RSDr %) and within-laboratory reproducibility (inter-day relative standard deviation RSD_R_%). Calibration curves exhibited a good degree of linearity (R^2^ ≥ 0.99, RF within ± 10% of the mean). LOQ, calculated assuming a signal/noise (S/N) ratio of 10, was 0.6 µg kg^−1^ while LOD (S/N ratio of 3) was 0.2 µg kg^−1^. Good performances were obtained also for accuracy (R% = 80–120%) and precision (RSD_r_ % and RSD_R_% < 10%).

### 4.4. Biological Analyses

#### 4.4.1. Immunological Analyses

Lysosomal membrane stability was evaluated in the haemocytes as neutral red retention time (NRRT) using the cationic probe neutral red (NR) (Sigma-Aldrich Co., St. Louis, MO, USA). Haemocytes were incubated on a glass slide with a freshly prepared NR working solution (2 µlL/mL filtered sea water from a stock solution of 20 mg NR dye dissolved in 1 mL of dimethyl sulfoxide) and microscopically examined (Olympus Co., Tokyo, Japan) at 20 min intervals to determine the time at which 50% of cells had lost into the cytosol the dye previously taken up by lysosomes.

Phagocytosis capacity assay was performed on haemolymph dispersed on glass slides and allowed to adhere for 15 min at 15 °C in the dark. Fluorescein-labelled Zymosan A bioparticles (Invitrogen, Carlsbad, CA, USA) were added at 10:1 target:haemocyte ratio. After 2 h incubation at 15 °C in the dark, uninternalized particles were removed by washing with physiological solution and slides were finally fixed in Beker’s fixative (+2.5% NaCl) and mounted in glycerol gelatine. Phagocytosis activity was expressed as percentage of cells that internalized at least three fluorescent particles (positive cells), observed under a fluorescence microscope (Olympus Co., Tokyo, Japan), after counting at least 200 cells for each sample.

For the analysis of granulocyte/hyalinocyte (G/H) ratio, aliquots of haemolymph were dispersed on glass slides and, after drying, fixed in Beker’s fixative (+2.5% NaCl). The slides were washed and stained with May Grunwald Giemsa (Sigma-Aldrich Co., St. Louis, MO, USA) before mounting in glycerol gelatine. Observations were carried out with a light microscope (1000×) (Olympus Co., Tokyo, Japan) and percentage of granulocytes was evaluated after counting at least 200 cells for each sample.

#### 4.4.2. Histological Analyses

Haematoxylin–eosin, neutral lipids and lipofuscin staining were performed on 8 µm cryostat sections of DG fixed in Beker’s fixative (+2.5% NaCl) for 15 min. For lipofuscin analysis, cryosections were stained by Schmrol reaction. For analyses of neutral lipids, cryosections were stained with the Oil Red O (ORO) (Sigma-Aldrich Co., St. Louis, MO, USA) method. Slides were mounted in glycerol gelatine and observed under light microscope (Olympus Co., Tokyo, Japan). For both lipofuscin and neutral lipids, five measurements were made on digestive tubules of each section (two sections for mussel). Quantification of staining intensity was performed with Image-Pro^®^ Plus 6.2 Analysis Software and then normalized to the area of digestive tubules.

#### 4.4.3. Biochemical Parameters and Total Oxyradical Scavenging Capacity Assay 

All chemicals used for biochemical analyses were purchased from Sigma-Aldrich Co. (St. Louis, MO, USA). The activity of peroxisomal ACOX was measured in DG homogenized in 1 mM sodium bicarbonate buffer (pH 7.6) containing 1mM EDTA, 0.1% ethanol, 0.01% Triton X-100 and centrifuged at 500 × *g* for 15 min at 4 °C. A coupled assay followed the oxidation of dichlorofluorescein-diacetate (DCF-DA) at 502 nm, catalysed by an exogenous horseradish peroxidase (HRP) in the presence of H_2_O_2_ produced by the oxidation of an exogenous substrate (palmitoyl-CoA) by the ACOX present in the sample. The reaction was performed in 0.5 M potassium phosphate buffer (pH 7.4), 2.2 mM DCF-DA, 40 µM sodium azide, 0.01% Triton X-100, 1.2 U mL^−1^ HRP in a final volume of 1 mL. After a pre-incubation at 25 °C for 5 min in the dark with an appropriate volume of sample, reactions were started by adding the substrate palmitoyl-CoA, at final concentrations of 30 µM; readings were carried out against a no-substrate blank at 502 nm (Varian spectrophotometer, model Cary 3).

AChE activity was spectrophotometrically assayed (Varian spectrophotometer, model Cary 3) in mussel haemolymph using the Ellman’s reaction, with acetylthiocholine and 5,5-dithiobis-2-nitrobenzoic acid (DTNB). 

The study of antioxidant responses in the DG was carried on through both the analyses of single antioxidant (the enzymatic activities of catalase, glutathione S-transferases, glutathione reductase, glutathione peroxidases and the levels of total glutathione) and the measurement of the total antioxidant capacity (TOSC assay towards peroxyl and hydroxyl radicals).

For enzymatic antioxidants, samples of DG were homogenized (1:5 w:v ratio) in 100 mM K-phosphate buffer (pH 7.5), with NaCl 2.5% and 0.1 mM phenylmethylsulphonyl fluoride (PMSF), 0.008 TIU mL^−1^ aprotinin, 1 µg mL^−1^ leupeptin, 0.5 µg mL^−1^ pepstatin as protease inhibitors. After centrifugation at 110,000 × *g* for 1 h at 4 °C, the supernatant (cytosolic fraction) was recovered and stored at −80 °C for enzymatic assays. The measurements were made with a Varian (model Cary 3) spectrophotometer at a constant temperature of 18 °C. Catalase (CAT) was measured by the decrease in absorbance at 240 nm (extinction coefficient, ε = 0.04 mM^−1^ cm^−1^) due to the consumption of hydrogen peroxide, H_2_O_2_ (12 mM H_2_O_2_ in 100 mM K-phosphate buffer pH 7.0). Glutathione S-transferases (GST) were determined at 340 nm using 1-chloro-2,4-dinitrobenzene (CDNB) as substrate. The assay was carried out in 100 mM K-phosphate buffer pH 6.5, 1.5 mM CDNB, 1 mM reduced glutathione (GSH) (ε = 9.6 mM^−1^ cm^−1^). Glutathione reductase (GR) was determined from NADPH (nicotinamide adenine dinucleotide phosphate) oxidation during the reduction of oxidized glutathione, GSSG (λ = 340 nm, ε = 6.22 mM^−1^ cm^−1^). The final assay condition was 100 mM K-phosphate buffer pH 7.0, 1 mM GSSG, and 60 mM NADPH. Glutathione peroxidases (GPx) activities were assayed in a coupled enzyme system where the GSSG, produced by GPx, was reduced to GSH by glutathione reductase with consumption of NADPH, which was monitored as decrease of absorbance at 340 nm (ε = 6.22 mM^−1^ cm^−1^). The reaction was performed in 100 mM K-phosphate buffer pH 7.5, 1 mM EDTA, 1 mM dithiothreitol, 2 mM GSH, 1 unit glutathione reductase, 0.24 mM NADPH, and 0.5 mM H_2_O_2_ (as substrate for Se-dependent GPx isoforms) or 0.8 mM cumene hydroperoxide (as substrate for the sum of Se-dependent and Se-independent forms). 

Levels of total glutathione (tGSH) in the DG were measured after homogenization (1:5, w/v ratio) in 5% sulphosalicilic acid with 4 mM EDTA. Samples were maintained for 45 min on ice for deproteinization and centrifuged at 37,000 × *g* for 15 min. The resulting supernatants were enzymatically assayed as previously reported [83]. Calibration was performed using reduced glutathione (GSH) standards.

The total oxyradical scavenging capacity (TOSC) assay measured the overall capability of cellular antioxidants to absorb different forms of artificially generated oxyradicals, thus inhibiting the oxidation of 0.2 mM α-keto-γ-methiolbutyric acid (KMBA) to ethylene gas [84]. Peroxyl radicals (ROO•) were generated by the thermal homolysis of 20 mM 2,2’-azo-bis-(2-methylpropionamidine)-dihydrochloride (ABAP) in 100 mM K-phosphate buffer, pH 7.4. Hydroxyl radicals (•OH) were produced by the Fenton reaction of iron-EDTA (1.8 mM Fe^3+^, 3.6 mM EDTA) plus ascorbate (180 mM) in 100 mM K-phosphate buffer. Under these conditions, the different oxyradicals produced quantitatively similar yields of ethylene in control reactions, thus allowing the comparison of the relative efficiency of cellular antioxidants toward a quantitatively similar radical flux. Ethylene formation in control and sample reactions was analysed at 12 min time intervals (total time: 96 min) by gaschromatographic analyses (Agilent Technologies, Santa Clara, CA, USA) and the TOSC values are quantified from the equation: TOSC = 100 − (ʃSA/ʃCA × 100), where ʃSA and ʃCA are the integrated areas calculated under the kinetic curves for samples (SA) and control (CA) reactions. For all the samples, a specific TOSC (normalized to content of protein) was calculated by dividing the experimental TOSC values by the relative protein concentration contained in the assay. 

Protein concentrations were measured according to the Lowry method, using bovine serum albumin (BSA) as standard.

#### 4.4.4. Measurement of Genotoxic Damage

The genotoxic damage was evaluated in mussels’ haemolymph through the Comet assay and the analysis of micronuclei frequency.

The DNA integrity was evaluated at molecular level, as single strand breaks (SB) by the Comet assay. The Comet assay was carried out on mussels’ haemocytes included in 1% low-melting-point agarose on glass slides, followed by treatment in lysis solution, DNA denaturation, electrophoresis and staining with 1 μg mL^−1^ 4′,6-diamidino-2-phenylindole (DAPI) (Sigma-Aldrich Co., St. Louis, MO, USA). One-hundred randomly selected “nucleoids” per slide, and two replicates per sample, were examined under fluorescence microscopy (200× magnification; Olympus Co., Tokyo, Japan), and the captured images (Image-Pro-Plus package) were analysed by Tritek CometScore™ software. The level of DNA fragmentation was estimated by measuring the percentage of DNA in the tail. 

At chromosomal level, the genotoxic effects were evaluated by the micronucleus (MN) test. MN frequency was measured in haemocytes, fixed in Carnoy’s solution (3:1 ethanol, acetic acid), dispersed on glass slides and stained with the fluorescent dye DAPI at 100 ng mL^−1^. For each specimen, 2000 cells with preserved cytoplasm were scored for the presence of MN, defined as round structures, smaller than 1/3 of the main nucleus diameter, on the same optical plan and clearly separated from it.

### 4.5. Statistical Analysis

Analysis of variance (ANOVA) was applied to all the determined parameters to test differences between experimental conditions (level of significance at *p* < 0.05). Cochram C was applied for testing homogeneity of variance, with appropriate mathematical transformation if necessary. The post hoc comparison Newman–Keuls was used to discriminate between concentrations means of AZA analogues in mussels’ tissues; the Dunnet test was used for post-hoc comparison to identify differences between control and exposed means of values (n = 5), for biological analyses. Statistical analyses were performed using R-cran software (http://www.R-project.org).

## 5. Conclusions

Our experiments demonstrated the bioaccumulation of specific AZA analogues (mainly AZA-54, 3-epi-AZA-7 and AZA-55) produced by a Mediterranean strain of *A. dexteroporum* in the tissues of exposed mussels, along with different rates of excretion of such toxins and relatively slow detoxification processes. AZAs bioaccumulation caused several biological responses, which can be recorded at different times of exposure and in some cases are still evident after three weeks of depuration. A clear modulation is evident in the immune system, with alteration of phagocytosis activity and haemocyte populations. Histopathological changes (i.e., tubule wall thinning) and neutral lipid reduction suggest that AZAs can affect the digestive activities with consumption of storage substances. Exposure to the toxic dinoflagellate seems to produce a limited involvement of oxidative metabolisms and no neurotoxicity effect, while genotoxic damage can be significant and relatively persistent. These first steps in the elucidation of early sublethal responses induced in mussels by the novel AZAs reveal that blooms of the Mediterranean *Azadinium dexteroporum* may represent a source of impairment for the health of natural population of bivalves. In addition, they provide indications of detectable biological responses in the exposed mussels, which could be an early indicator of AZA contamination associated with the human consumption of seafood. 

## Supplementary Materials

The following are available online at https://www.mdpi.com/1660-3397/17/10/595/s1, Table S1: Chromatographic conditions utilized in LC-MS/MS analysis, Table S2: Ion-spray source MS parameters, Table S3: Mass transitions and collision energy used for AZAs determination in multiple reaction monitoring (MRM) experiments.
