# Supplementary material for: Biological Effects of the Azaspiracid-Producing Dinoflagellate Azadinium dexteroporum in Mytilus galloprovincialis from the Mediterranean Sea
Source: Mar Drugs. 2019 Oct 22;17(10):595. doi: 10.3390/md17100595 (PMC6835248; doi:10.3390/md17100595)
Supplement: Supplementary file 1 [file marinedrugs-17-00595-s001.pdf]

SUPPLEMENTARY MATERIALS

Biological Effects of the Azaspiracid-Producing Dinoflagellate *Azadinium dexteroporum* in *Mytilus galloprovincialis* from the Mediterranean Sea

Maria Elisa Giuliani [1,†], Stefano Accoroni [1,2,†], Marica Mezzelani [1,†], Francesca Lugarini [1], Simone Bacchiocchi [2], Melania Siracusa [2], Tamara Tavoloni [2], Arianna Piersanti [2], Cecilia Totti [1], Francesco Regoli [1], Rachele Rossi [3], Adriana Zingone [4] and Stefania Gorbi [1,*]

1 Dipartimento di Scienze della Vita e dell'Ambiente, Università Politecnica delle Marche, Via Brecce Bianche, 60131 Ancona, Italy
2 Istituto Zooprofilattico Sperimentale Umbria e Marche, Via Cupa di Posatora, 3, 60131 Ancona, Italy
3 Istituto Zooprofilattico Sperimentale del Mezzogiorno, Via Salute 2, 80055 Portici (NA), Italy
4 Dipartimento di Ecologia Marina Integrata, Stazione Zoologica Anton Dohrn, Villa Comunale, 80121 Napoli, Italy
* Correspondence: s.gorbi@univpm.it; Tel.: +39-071-220-4142
† These authors equally contributed to this work.

Table S1. Chromatographic conditions utilised in LC-MS/MS analysis.

**Column**	Waters X-Bridge C18, 150 mm x 3mm; 5µm		
**Run time**	27 min		
**Column temperature**	40 °C		
**Injection volume**	10 µl		
**Flow rate**	0.4 ml min $^{-1}$		
**Gradient**	**Time** (min)	**Mobile phase A** (%)	**Mobile phase B** (%)
	0	90	10
	2	90	10
	13	10	90
	18	10	90
	21	90	10
	27	90	10

Table S2. Ionspray source MS parameters.

**Turbo IonSpray source**	
Temperature (**TEM**)	600 °C
Ionspray voltage (**IS**)	5000 V
Curtain gas (**CUR**)	20 psi
Nebulizer Gas (**GS1**)	60 psi
Auxiliary Gas (**GS2**)	40 psi
CAD	Medium

Table S3. Mass transitions and collision energy used for AZAs determination in Multiple Reaction Monitoring (MRM) experiments.

Azaspiracids AZAs	Precursor Ion (m/z)	Product Ion (m/z)	CE (eV)
**AZA-1**	[a]842	824	55
		672	70
**AZA-54**	[a]870	852	55
		672	70
**3-epi-AZA-7**	[a]858	840	55
		672	70
**AZA-58**	[a]828	810	55
		672	70
**AZA-55**	[a]868	850	55
		670	70

[a]Present as $[M + H]^+$; CE= energy collision
